# Pax3 Stimulates p53 Ubiquitination and Degradation Independent of Transcription

**DOI:** 10.1371/journal.pone.0029379

**Published:** 2011-12-28

**Authors:** Xiao Dan Wang, Sarah C. Morgan, Mary R. Loeken

**Affiliations:** Section on Developmental and Stem Cell Biology, Department of Medicine, Joslin Diabetes Center, Harvard Medical School, Boston, Massachusetts, United States of America; Instituto de Medicina Molecular, Portugal

## Abstract

**Background:**

Pax3 is a developmental transcription factor that is required for neural tube and neural crest development. We previously showed that inactivating the p53 tumor suppressor protein prevents neural tube and cardiac neural crest defects in *Pax3*-mutant mouse embryos. This demonstrates that Pax3 regulates these processes by blocking p53 function. Here we investigated the mechanism by which Pax3 blocks p53 function.

**Methodology/Principal Findings:**

We employed murine embryonic stem cell (ESC)-derived neuronal precursors as a cell culture model of embryonic neuroepithelium or neural crest. Pax3 reduced p53 protein stability, but had no effect on *p53* mRNA levels or the rate of p53 synthesis. Full length Pax3 as well as fragments that contained either the DNA-binding paired box or the homeodomain, expressed as GST or FLAG fusion proteins, physically associated with p53 and Mdm2 both *in vitro* and *in vivo*. In contrast, *Splotch* Pax3, which causes neural tube and neural crest defects in homozygous embryos, bound weakly, or not at all, to p53 or Mdm2. The paired domain and homeodomain each stimulated Mdm2-mediated ubiquitination of p53 and p53 degradation in the absence of the Pax3 transcription regulatory domains, whereas *Splotch* Pax3 did not stimulate p53 ubiquitination or degradation.

**Conclusions/Significance:**

Pax3 inactivates p53 function by stimulating its ubiquitination and degradation. This process utilizes the Pax3 paired domain and homeodomain but is independent of DNA-binding and transcription regulation. Because inactivating p53 is the only required Pax3 function during neural tube closure and cardiac neural crest development, and inactivating p53 does not require Pax3-dependent transcription regulation, this indicates that Pax3 is not required to function as a transcription factor during neural tube closure and cardiac neural crest development. These findings further suggest novel explanations for PAX3 functions in human diseases, such as in neural crest-derived cancers and Waardenburg syndrome types 1 and 3.

## Introduction

Understanding how regulators of embryonic development function on a molecular level is a major objective of developmental biology. Pax3, a member of the mammalian Pax family of developmental regulators, is expressed in the neuroepithelium, neural crest, and somitic mesoderm [1], [2], [3]. One hundred percent of mouse embryos that are homozygous for the mutant *Pax3* allele, *Splotch* (*Pax3^Sp/Sp^*) develop neural tube defects (NTD), cardiac outflow tract defects (COTD), and fail to form skeletal muscle, indicating that Pax3 is essential for formation of these structures [4], [5], [6]. In humans, Waardenburg syndrome (WS) types 1 and 3 is an autosomal dominant condition that is caused by *PAX3* mutations and affects neural crest-derived structures, [7]. Pax proteins are characterized by the presence of a paired box DNA-binding element [1]. Some of the Pax proteins, including Pax3, contain a paired-type homeodomain that also binds to DNA, and a conserved octapeptide [1]. It has traditionally been accepted that Pax3 regulates developmental processes by operating as a transcriptional regulator because: (i) Pax3 contains sequence-specific DNA-binding domains that are capable of directing *trans*-activation [8], [9], [10]; (ii) the protein product of the mutant *Splotch Pax3* allele is *trans*-activation defective [11]; and (iii) several genes have been identified that are directly or indirectly regulated by Pax3 [12], [13], [14], [15], [16], [17], [18], [19], [20], [21], [22], [23]. However, exactly how Pax3 regulates formation of the neural tube and neural crest-dependent structures has not yet been determined.

Apoptotic cells are observed in embryos expressing nonfunctional *Pax3* alleles at sites where normal Pax3-expressing neuroepithelial and cardiac neural crest cells are located in w.t. embryos [24], [25], [26]. This suggests that the ensuing NTD and COTD result from depletion of progenitor cells that are necessary to populate these structures. We showed that inactivation of p53 through germ-line mutation or chemical inhibition prevented the NTD, exencephaly and spina bifida, and COTD that are characteristic of *Pax3^Sp/Sp^* embryos, as well as associated apoptosis, in embryos expressing nonfunctional *Pax3* alleles [25], [27]. This indicates that Pax3 is not required in neuroepithelium and neural crest to regulate genes that direct morphogenesis or migration, but that it is required to block p53-dependent processes that lead to apoptosis. This contrasts with the role of Pax3 in skeletal muscle development where it serves as an upstream regulator of myogenic gene expression [16], [17]. p53 protein, but not mRNA, was increased in *Pax3^Sp/Sp^* embryos, suggesting that Pax3 blocks p53 function by inhibiting p53 protein synthesis, stability, or both [27]. However, the precise mechanism by which Pax3 regulates steady state levels of p53 protein, and whether it involved Pax3 functioning as a transcription factor, has not been determined.

Further study of the molecular mechanism by which Pax3 regulates p53 can be facilitated by a cell culture model of developing neuroepithelium and neural crest. Murine embryonic stem cells (ESC) can be induced to form neuronal precursors that express genes that are characteristic of neuroepithelium, including *Pax3* [28]. Thus, if expression of Pax3 causes a reduction in steady-state levels of p53 protein, differentiating ESC would be a valid cell culture model to study the mechanism by which Pax3 blocks p53 function in embryonic neuroepithelium and neural crest.

## Results

### p53 protein is negatively regulated by Pax3 in ESC

**Figure 1:**
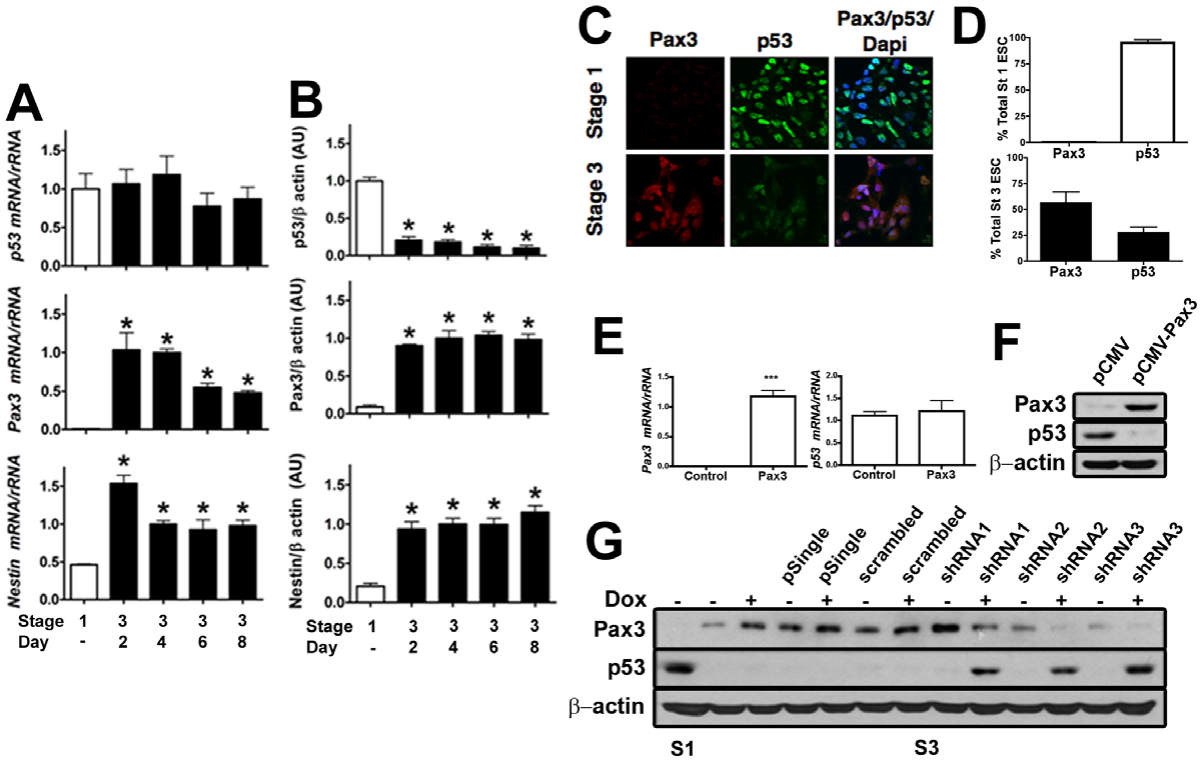
Pax3 negatively regulates p53 protein, but not mRNA levels in ESC just as in mouse embryos. (A) Real time RT-PCR of *p53*, *Pax3*, and *Nestin* mRNA in stage 1 (open bars) and stage 3, days 2–8 (solid bars) ESC. *Nestin* mRNA is expressed in neuroepithelium and in ESC-derived neuronal precursors [29], [30] and served as a control for a marker of neuroepithelial neuronal precursors. Each mRNA was normalized to rRNA. In (A), (B), and (D) values represent the mean ± SEM (n = 3 culture dishes). *p<0.01 vs. stage 1. (B) Quantification (band intensity in arbitrary units) of immunoblots of p53, Pax3, and Nestin normalized to β-actin in stage 1 and stage 3 ESC harvested on days as indicated in (A). (C) Indirect immunofluorescence of p53 (green) and Pax3 (red) in stage 1 and stage 3 ESC. Cells were counterstained with DAPI (blue) to visualize nuclei. Cells incubated with secondary antibodies alone generated no detectable signals (not shown). (D) Percent Pax3 or p53 positive cells in stage 1 and stage 3 ESC. Values represent the mean ± SEM (n = 10 fields). (E) Real time RT-PCR of *Pax3* and *p53* mRNA in stage 1 ESC transfected with empty vector (Control), or vector encoding w.t. Pax3. Each mRNA was normalized to rRNA. ***p<0.0001 vs. control cells. (F) Immunoblot of Pax3 or p53 in stage 1 ESC stably transfected with empty pCMV vector or pCMV-Pax3. (G) Immunoblot of Pax3 or p53 in stage 1 or stage 3 ESC. Stage 3 ESC were untransfected, or transfected with empty shRNA vector (pSingle), pSingle expressing a scrambled shRNA sequence (scrambled), and 3 different Pax3 shRNA sequences. Stage 3 cultures were treated or not with doxycycline during days 4–6.

We first investigated whether abundance of p53 protein, but not mRNA, was inversely related to abundance of Pax3 in ESC as in mouse embryos. Murine ESC were grown as undifferentiated cultures (stage 1), or were induced to form neuroepithelial-like neuronal precursors (stage 3) using established methods [29]. There was no difference in abundance of *p53* mRNA between stage 1 and stage 3 (Figure 1 A). In contrast, *Pax3* mRNA was undetectable in stage 1 ESC but was significantly increased during stage 3. *Nestin* mRNA, which is expressed in neuroepithelium *in vivo* and in mESC-derived neuronal precursors [29], [30] increased in stage 3 ESC. In contrast to *p53* mRNA, p53 protein significantly decreased during ESC differentiation, while Pax3 protein increased in parallel to *Pax3* mRNA (Figure 1 B). Nestin protein levels also significantly increased in stage 3 ESC. Immunofluorescence using antibodies against p53 or Pax3 further supported that p53 and Pax3 protein abundance are inversely related in undifferentiated and differentiating ESC (Figure 1 C, D). Thus, in ESC, just as in mouse embryos, p53 protein, but not mRNA, is inversely related to production of Pax3.

To test whether Pax3 was responsible for the decrease in p53 protein in differentiating ESC the effects of expressing Pax3 in stage 1 ESC, and of knocking down expression of Pax3 in stage 3 ESC, on p53 were examined. Transfecting ESC with a Pax3 expression vector showed that constitutive expression of Pax3 had no effect on *p53* mRNA (Figure 1 E) but was sufficient to suppress p53 protein in stage 1 ESC (Figure 1 F). Conversely, knocking down Pax3 using an inducible shRNA in stage 3 ESC increased p53 protein (Figure 1 G). These results demonstrate that Pax3, and not the process of differentiation *per se*, is responsible for the decrease in p53 in stage 3 ESC.

### Pax3 stimulates p53 ubiquitination and degradation

**Figure 2:**
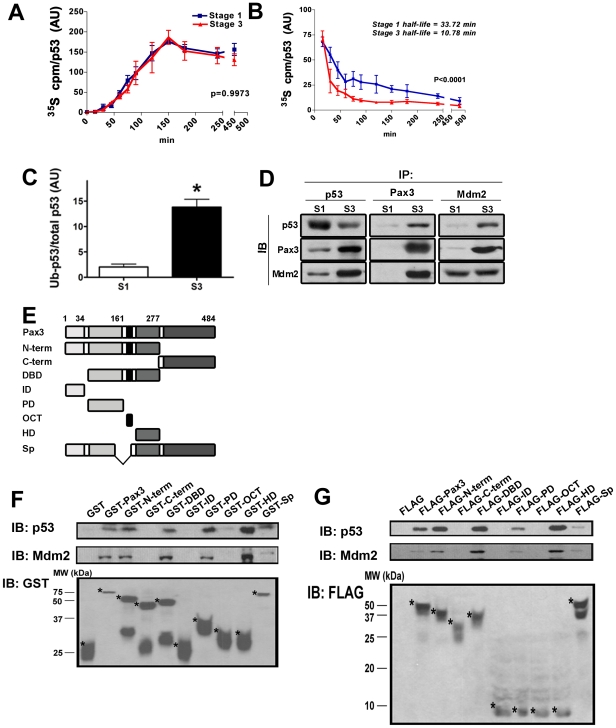
Pax3 stimulates p53 degradation and ubiquitination and physically associates with p53 and Mdm2. (A) Pulse labeling with ^35^S-met to determine the rate of p53 synthesis in stage1 and stage 3 ESC. Quantitation of ^35^S-p53 is described in the supporting online material. (B) Pulse-chase labeling to determine the t_12_ of p53 in stage 1 and stage 3 ESC. (C) Quantitation of ubiquitinated p53/total p53 in stage 1 and stage 3 ESC following immunoprecipitation of p53 and immunoblotting using anti-ubiquitin or anti-p53 antibodies. *p<0.05 vs. stage 1. (D) Whole cell extracts of stage 1 or stage 3 ESC were immunoprecipitated using antibodies against p53, Pax3 or Mdm2, and then immunoblotted using antibodies against p53, Pax3, and Mdm2. (E) Schematic diagram of full-length w.t. Pax3, Pax3 structural domains, and a *Splotch* Pax3 (Sp) protein product that were expressed as GST and FLAG fusion proteins. N-term, amino-terminus of Pax3 through the homeodomain; C-term, carboxy-terminus distal to the homeodomain (including the *trans* activation domain); DBD, DNA-binding domain (PD through HD); ID, the trans-activation inhibitory domain (amino-terminal to the PD); PD, paired domain; OCT, octapeptide (carboxy-terminal of the PD to amino-terminal of the HD); and HD, homeodomain. The Splotch cDNA deletes exon 4 and lacks coding sequence for part of the PD and the OCT but retains the HD. Numbers refer to amino acid positions of w.t. Pax3. (F) Immunoblot using antibodies against p53 (upper panel), Mdm2 (middle panel), or GST following incubation of whole cell lysates from stage 1 ESC with GST-Pax3 fusion proteins linked to glutathione-sepharose beads. (G) Immunoblot using antibodies against p53 (upper panel), Mdm2 (middle panel), or FLAG following incubation of whole cell lysates from stage 1 ESC that had been transiently transfected with plasmids encoding FLAG-tagged Pax3 fusion proteins with antibody against FLAG linked to M2 beads.

Treatment of lung carcinoma cells transfected with a *PAX3* expression plasmid with cycloheximide suggested that Pax3 stimulates p53 degradation [31], however, whether synthesis of p53 or synthesis of a regulator of p53 turnover was also inhibited by cyclohexamide was not determined. To examine whether the decrease in p53 protein in ESC-derived neuronal precursors was due to a reduction in protein synthesis, stability, or both, newly synthesized p53 was pulse labeled with ^35^S-met. The rate of incorporation of ^35^S-met into p53 demonstrated that p53 protein synthesis was not reduced in stage 3 ESC (Figure 2 A). However, assay of ^35^S-met-labeled p53 followed by a chase with unlabeled met demonstrated that the t_1/2_ of p53 is reduced approximately 3-fold in stage 3 ESC compared to stage 1 ESC (Figure 2 B). p53 degradation is stimulated by association with Mdm2 and activation of Mdm2 ubiquitin ligase activity [32]. To test whether decreased stability of p53 in stage 3 ESC might be due to increased ubiquitination, ubiquitinated p53 relative to total p53 was assayed by immunoblot. The amount of p53 that was ubiquitinated was significantly increased in stage 3 ESC (Figure 2 C), suggesting that Pax3 stimulates p53 degradation by promoting its ubiquitination.

Although it is possible that Pax3 could regulate expression of genes whose products participate in p53 ubiquitination, we considered that a more rapid modulation of p53 degradation could be effected if Pax3 physically associates in a complex containing p53 and Mdm2. To study this, we immunoprecipitated p53, Pax3, or Mdm2 and examined protein associations by immunoblot. As shown in Figure 2 D, Pax3 and p53 associated with each other, particularly using extracts from stage 3 ESC, and Pax3 also associated with Mdm2. As expected, Mdm2 co-immunoprecipitated with p53, although more of p53 and Mdm2 were associated with each other using extracts from stage 3 ESC than from stage 1 ESC. Inasmuch as p53 is less abundant in stage 3 than in stage 1 ESC, it is possible that Pax3 promotes association of p53 with Mdm2.

We next investigated which structural domains of Pax3 are responsible for association with p53 or Mdm2. As diagrammed in Figure 2 E, the structural domains of Pax3 that have been previously identified include an N-terminal transcription inhibitory domain (ID), the paired domain (PD), a conserved octapeptide (OCT), the paired-type homeodomain (HD), and a C-terminal *trans*-activation domain (TAD). The PD and HD are each independent DNA-binding domains [3], [9], [10] that bind to DNA with higher affinity together than when only one of the domains is bound together [8], the OCT is necessary for homodimerization [11], and the ID and TAD possess transcription inhibition and transcription activation activities, respectively [33]. We constructed plasmids to express glutathione-S-transferase- (GST) and FLAG-tagged proteins fused with full-length w.t. Pax3, or fragments containing various Pax3 structural domains. Additionally, we constructed plasmids to express GST or FLAG fused with the least defective of the proteins encoded by the mutant *Pax3* allele, *Splotch*. The *Splotch* mutation disrupts the splice acceptor site of exon 4, resulting in four aberrantly spliced transcripts [11], [34], [35]. Three of the predicted protein products cause frame-shifts beginning in the PD, but the least defective deletes 45 amino acids that include part of the PD and the OCT (Figure 2 E).

GST fusion proteins were incubated with extracts from stage 1 ESC to determine which Pax3 structures can associate with p53 or Mdm2 *in vitro* (Figure 2 F), and plasmids encoding FLAG fusion proteins were transiently transfected into stage 1 ESC to determine which Pax3 structures can associate with p53 or Mdm2 in intact cells *in vivo* (Figure 2 G). All fusion proteins containing either the PD or HD associated with p53 and Mdm2 both *in vivo* and *in vitro*. Notably, the PD and HD were each able to associate with p53 and Mdm2 in the absence of the other DNA-binding domain. p53 associated with *Splotch* Pax3 at levels comparable to that of w.t. Pax3 *in vitro*, but only weakly *in vivo*. The weak association of *Splotch* Pax3 *in vivo*, despite the presence of the HD, which can associate with p53 in the absence of other Pax3 structural domains, suggests that the part of the PD and OCT that are deleted in *Splotch* Pax3 are necessary to prevent interference by the N-terminal transcription inhibitory domain or the C-terminal trans-activation domain for association of the HD with p53. Mdm2 only weakly associated with *Splotch* Pax3 both *in vitro* and *in vivo*.

### The Pax3 paired domain and homeodomain stimulate Mdm2-mediated ubiquitination of p53 and p53 degradation

**Figure 3:**
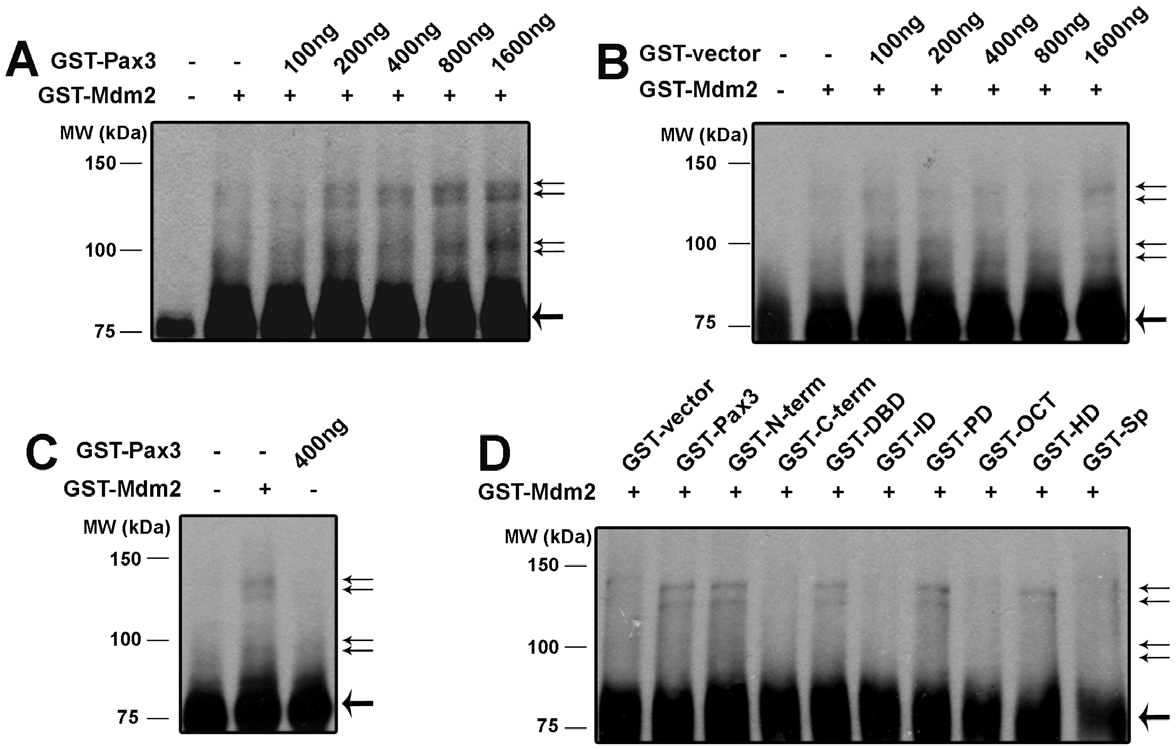
Pax3 stimulates Mdm2-mediated ubiquitination of p53 *in vitro*. (A) *In vitro* ubiquitination reactions of GST-p53 with GST-Mdm2 (150 ng), and 0–1600 ng GST-Pax3. Ubiquitination was assayed by immunoblot using 53 antibodies. The position of GST-p53 is indicated by a heavy arrow, and the positions of ubiquitinated p53 are indicated by narrow arrows. (B) *In vitro* ubiquitination reactions as in (A) except that GST without any Pax3 coding sequences was used. (C) *In vitro* ubiquitination reactions performed with only GST-Mdm2 or 400 ng GST-Pax3. (D) *In vitro* ubiquitination reactions of GST-p53 with GST-Mdm2 and 400 ng GST fusion proteins of w.t. Pax3, Pax3 structural domains, or *Splotch* Pax3. (The GST-p53 and GST-Mdm2 vectors used here encoded murine p53 and Mdm2, although similar results were obtained using human p53 and Mdm2 fusion proteins (data not shown).)

The physical association of Pax3 with p53 and Mdm2 suggested that Pax3 might regulate p53 ubiquitination. To test this, ubiquitination of GST-p53 by GST-Mdm2 in the presence or absence of GST-Pax3 was assayed *in vitro*. Ubiquitination of GST-p53 was stimulated by GST-Pax3 in a dose-dependent fashion (Figure 3 A). This activity was dependent on Pax3 structures, as GST alone did not stimulate p53 ubiquitination (Figure 3 B). GST-Pax3 did not stimulate p53 ubiquitination in the absence of GST-Mdm2 (Figure 3 C), demonstrating that Pax3 was not itself an ubiquitin ligase, but that it stimulated ubiquitin ligase activity of Mdm2.

To determine which structural domain(s) of Pax3 are responsible for stimulation of p53 ubiquitination, *in vitro* ubiquitination of GST-p53 by GST-Mdm2 with the addition of each of the GST fusion proteins containing Pax3 structural domains was examined. Each of the structural domains that are capable of complex formation with p53 and Mdm2 stimulated ubiquitination of GST-p53, although the PD appeared to be more potent than the HD (Figure 3 D). Notably, GST-*Splotch* Pax3 did not increase ubiquitination of GST-p53.

**Figure 4:**
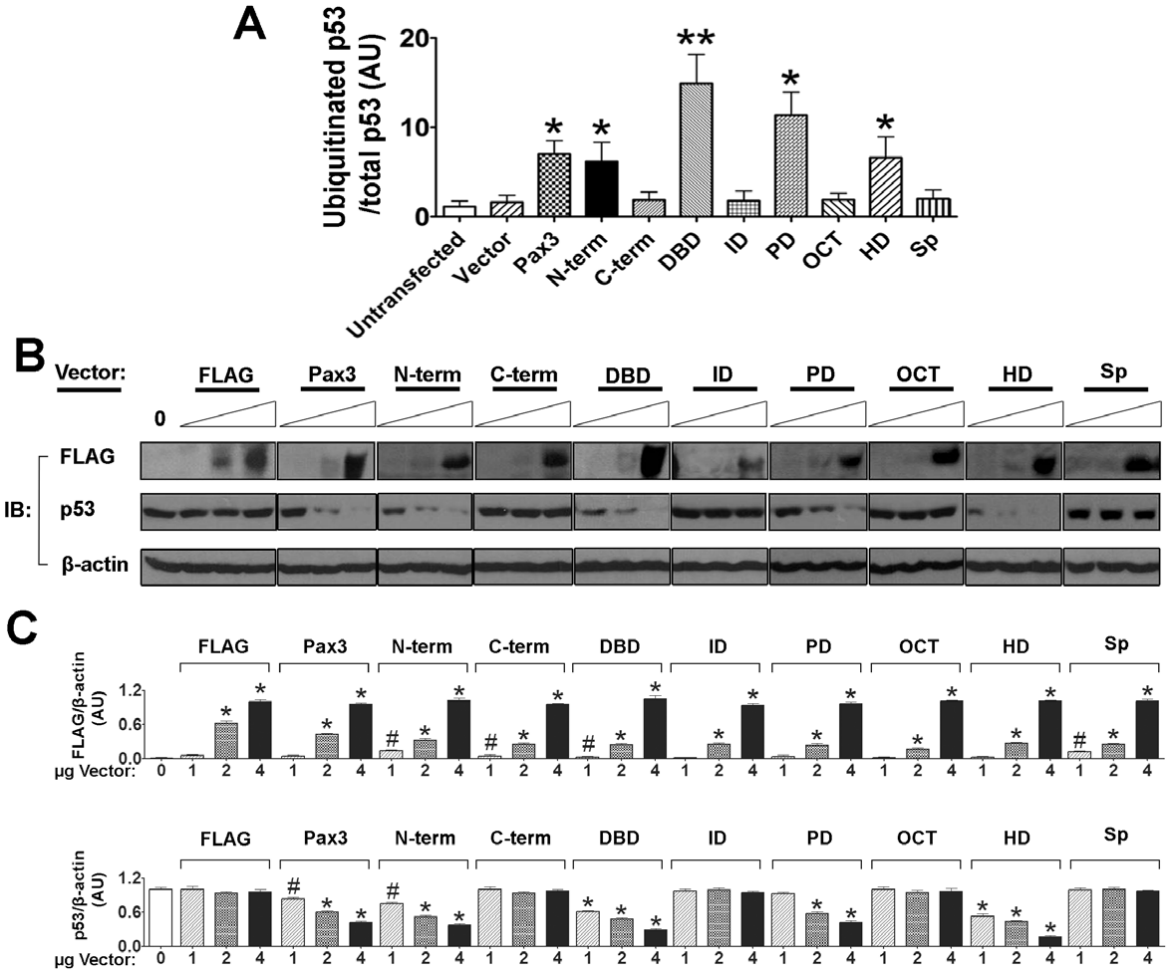
The Pax3 domains that associate with p53 and Mdm2 stimulate p53 ubiquitination and down regulation *in vivo*. (A) Quantitation of i*n vivo* ubiquitination of p53 in stage 1 ESC that were untransfected, or transiently transfected with 4 µg FLAG-tagged vectors encoding w.t. Pax3, Pax3 structural domains, or *Splotch* Pax3. *p<0.05, **p<0.01. (B) Immunoblot analyses of whole cell extracts from Stage 1 ESC that were untransfected, or transiently transfected with 1–4 µg FLAG-tagged vectors as in (A) using antibodies against FLAG, p53, or β-actin. (C) Quantitation of FLAG or p53 relative to β-actin from three replicate transfected culture dishes assayed as in (B).

To test whether the same Pax3 structural domains that can stimulate Mdm2-mediated p53 ubiquitination *in vitro* can stimulate p53 ubiquitination and degradation upon expression in ESC, plasmids encoding FLAG fusion proteins were transiently transfected into stage 1 ESC. As shown in Figure 4 A, the Pax3 structures that contain the PD or the HD, except for *Splotch* Pax3, stimulated p53 ubiquitination *in vivo*, just as they did *in vitro*. Transfecting increasing concentrations of plasmids encoding FLAG fusion proteins caused a dose-dependent decrease in steady state levels of p53 only if they encoded Pax3 structures that were capable of stimulating p53 ubiquitination (Figure 4 B, C). Because each of the PD and HD were capable of stimulating p53 ubiquitination and down regulation when they were expressed in the absence of the C-terminal trans-activation domain or the N-terminal transcription inhibitory domain, this indicates that down regulation of p53 does not require Pax3 to function as a transcriptional regulator. There was no effect of increasing concentrations of FLAG-*Splotch* Pax3 on p53 steady state levels. This is not due to decreased stability of the *Splotch* Pax3 protein, because steady-state levels of FLAG-*Splotch* Pax3 were similar to those of FLAG-w.t. Pax3 (Figure 4 B, C). Instead, the failure of *Splotch* Pax3 to decrease p53 levels appears to be due to defective association of *Splotch* Pax3 with p53 and Mdm2, and failure to stimulate Mdm2 ubiquitin ligase activity.

## Discussion

It has long been recognized that Pax3 is essential for formation of the neural tube and neural crest-dependent structures. Until now, it has been believed that the activity of Pax3 as a DNA-binding transcription factor is responsible for these functions, and that the phenotype of *Pax3^Sp/Sp^* embryos is due to loss of transcription factor activity of *Splotch* Pax3 [11], [35]. Our previous studies demonstrated that neural tube closure, cardiac neural crest migration, and cardiac outflow tract septation proceeds normally in *Pax3^Sp/Sp^* and *Pax3*-null embryos as long as p53 is inactivated [25], [27], demonstrating that Pax3 is required for these processes only to inactivate p53. The results presented here demonstrate that Pax3 inactivates p53 by stimulating its degradation and that stimulation of p53 degradation occurs independent of Pax3 binding to DNA and regulation of transcription. Therefore, while Pax3 may, in addition, regulate gene expression during neural tube and neural crest development, our results indicate that Pax3 is required in order to effect neural tube closure and neural crest-dependent cardiac outflow tract septation only to stimulate p53 ubiquitination and degradation independent of transcription. Moreover, these findings show that the mutant *Splotch Pax3* allele is defective in associating with p53 and Mdm2 and fails to stimulate p53 ubiquitination and down regulation. Because stimulation of p53 degradation by w.t. Pax3 occurs independent of its activity as a transcription factor, this indicates that *Splotch* Pax3 is functionally null, not because it is defective as a transcription factor, but because it fails to effectively complex with p53 and Mdm2 and to stimulate Mdm2-mediated ubiquitination of p53.

Associations of the Pax3 PD and HD with other proteins, including the retinoblastoma tumor suppressor protein (Rb), Msx1, Mox1 and Mox2, and Ets have been reported [36], [37], [38], [39]. However, each of these associations functioned to block Pax3 binding to DNA and activating transcription. In contrast, the association of the Pax3 DNA-binding domains with p53 and Mdm2 that are reported here confers an activity on these domains separate from gene regulation.

Additionally, our findings suggest that human diseases associated with PAX3 may be explained by insufficient or excessive p53 degradation. For example, almost all of the 76 different *PAX3* mutations that have been identified in WS type 1 and type 3 are localized to the PD, the OCT, or the HD (Figure S1 and Table S6). Because these mutations interfere with the transcription factor activity of PAX3, it has been widely accepted that altered expression of PAX3 target genes is responsible for the WS phenotype. However, our results predict that mutation of these PAX3 domains ought to also impair stimulation of HDM2-mediated ubiquitination of p53. Thus, failure to block p53-dependent processes, rather than altered expression of PAX3 target genes, may be responsible for the WS phenotype. It is intriguing to speculate that WS PAX3 proteins compete with w.t. PAX3 for associating with p53 or HDM2 and behave as trans-dominant mutants. This could explain why the WS *PAX3* alleles, which appear to be loss-of-function mutations, cause an autosomal dominant disease. Further research will be necessary in order to determine whether this is the case. On the flip side, *PAX3* over expression occurs in many neural crest or neuroectodermal tumors such as melanoma, neuroblastoma, and Ewing's sarcoma [40], [41], [42], [43], [44]. The *p53* gene is rarely deleted or mutated in these tumors [45], [46]. Thus, p53 loss of function may be accomplished by physical interaction with PAX3, and this may be crucial to the oncogenesis of these tumors. Other Pax proteins have oncogenic potential, as indicated by fibroblast transformation [47]. However, only Pax5 has been shown to regulate p53, and this was by direct transcription inhibition [48]. Whether down regulation of p53 by either transcriptional or post-transcriptional mechanisms is a general property of the Pax family remains to be determined.

Our conclusions may appear audacious given the existing dogma that Pax3 regulates neural tube closure and cardiac neural crest development by virtue of its activity as a transcription factor. However, reexamination of the existing literature in light of our findings can reveal a new paradigm of the mechanism by which Pax3 regulates neural tube and neural crest development. In particular, there are several genes, including two identified by us, whose expression is increased or decreased by Pax3 [12], [13], [14], [15], [16], [17], [18], [19], [20], [21], [22], [23]. Nonetheless, heretofore there is little functional evidence that Pax3 directly regulates any of these putative target genes and that they are mechanistically involved in neural tube closure or neural crest development. The only gene for which there is functional evidence is *Msx2*, whose expression is negatively regulated by Pax3 in the neural tube and neural crest [19]. *Msx2* loss-of-function (*Msx2^−/−^*) rescued COTD and embryonic lethality in *Pax3^Sp/Sp^* embryos, although NTD were not rescued [19]. This evidence notwithstanding, the putative Pax3 binding site within the *Msx2* promoter is low affinity [8], suggesting that Pax3 might not directly regulate *Msx2* under physiological conditions. There is evidence that *Msx2* expression is upregulated along with *p53* [49], suggesting that *Msx2* might be a direct or indirect target of p53. Thus, while Pax3 can directly regulate *Msx2* under experimental conditions, effects of Pax3 on *Msx2* expression in embryonic cardiac neural crest may be indirect and mediated by altered p53 levels. The failure of *Msx2* deletion to rescue NTD in *Pax3^Sp/Sp^* embryos indicates that *Msx2* is not a functional target of Pax3 in the neural tube, or that its downregulation is not sufficient to mediate effects of Pax3 on neural tube closure.

Interestingly, it was recently reported, using conditional deletion of *Pax3* in premigratory and/or migratory neural crest, that Pax3 is only required for expression in early premigratory and migratory cardiac neural crest [50]. This is consistent with our findings, using pifithrin-α, that inhibition of p53 by Pax3 is only required during approximately the first 4 hours after the onset of *Pax3* expression on E8.5 in order for normal cardiac neural crest migration and outflow tract septation to occur [25]. Thus, while Pax3-linked reporter gene expression can be detected in cardiac neural crest cells at least through the 35 somite stage (approximately E9.5) [25], it is only required to block p53-dependent processes that are required for subsequent outflow tract septation in early premigratory and migratory cardiac neural crest cells.

Our findings lead to the overarching question of why it is necessary for Pax3 to functionally inactivate p53 during embryonic development. Studies using ESC or generation of induced pluripotent stem (iPS) cells have indicated that p53 is activated during ESC differentiation, and that activation of p53 inhibits self-renewal and promotes differentiation [51], [52], [53], [54], [55]. Thus, it may be necessary for Pax3 to titrate the activity of p53 once embryonic cells start to differentiate along a neural lineage in order to prevent premature loss of proliferative capability and multipotency until a critical cell mass or cellular localization is achieved.

## Materials and Methods

### Ethics Statement

Mouse embryos used for recovery of RNA for generation of p53 and Mdm2 expression plasmids were obtained from pregnant mice on E10.5 using procedures that are approved by the Joslin Diabetes Center Institutional Animal Care and Use Committee under Protocol #92-06.

### Embryonic Stem Cell Culture

Mouse D3 ESC (ATCC) were cultured and induced to form neuronal precursors as described [29], except that 0.5 µM retinoic acid (Sigma) was added to embryoid body cultures. Briefly, cells were grown as undifferentiated, monolayer cultures (referred to as stage 1) on 0.1% gelatin-coated tissue culture dishes (without mouse embryo fibroblasts) in DMEM (Invitrogen) containing 10% fetal calf serum (Atlanta Biologicals), 110 µM β-mercaptoethanol (Sigma), 1000 units/ml of leukocyte inhibitory factor (LIF), 2 mM glutamine, 1× nucleosides, 1× nonessential amino acids (all from Millipore), and 1× penicillin/streptomycin (Invitrogen). To induce differentiation to neuronal precursors, stage 1 cultures were trypsinized, and were induced to form embryoid bodies (referred to as stage 2) in bacterial grade culture dishes containing stage 1 media with 0.5 µM retinoic acid, but without LIF, for 4 days, then were transferred to tissue culture dishes in DMEM:F12 media (Invitrogen) containing fibronectin (Becton Dickenson), insulin, transferrin, and selenium (Sigma) for up to 4 days (referred to as stage 3). All experiments were performed using cells that had undergone fewer than 40 passages.

### Plasmid Constructions

Short hairpin RNA (shRNA) sequences targeting *Pax3* mRNA were designed using the shRNA Sequence Designer (Clontech), and a scrambled sequence was designed using BLOCK-iT RNAi Designer (Invitrogen). Three *Pax3*-targeted shRNA sequences and the scrambled sequence (Table S1) were inserted into the Xho1 and HindIII sites of pSingle-tTS-shRNA (Clontech). Presence of inserts was determined by restriction digestion with MluI (Promega).

GST fusion plasmids encoding w.t. Pax3, the amino-terminus, carboxy-terminus, DBD, ID, PD, OCT, and HD (see Figure 2 E) were generated as described [8], except using primers and PCR conditions shown in Table S2. The *Splotch* Pax3 cDNA was generated by PCR of GST-w.t. Pax3 to delete exon 4 (nt 839–973) which encodes the C-terminal 14 amino acids of the PD and the OCT; this cDNA encodes the only in-frame *Pax-3* mRNA produced in *Sp/Sp* mutants [34], [35]. GST fusion proteins encoding murine Mdm2 and p53 coding were generated using cDNA obtained from E 10.5 mouse embryos.

FLAG fusion vectors were constructed by removing the Pax3 coding sequences from the GST fusion plasmids with BamHI and EcoRI and inserting them into the BamHI and EcoRI sites of pCMV-Tag2C (Stratagene). The plasmids were grown in DH5α competent *E. coli* (Invitrogen) and purified using Qiagen plasmid preparation kits (Qiagen, Inc.).

All PCR were performed using Taq ready mix (Sigma, St Louis, MO), except the PCR to generate the Splotch Pax3 internal deletion, in which *PfuUltr* High Fidelity DNA polymerase (Stratagene, La Jolla, CA) was used. The PCR products were inserted into pGEX-3X (GE healthcare, Piscataway, NJ) that had been digested with BamHI and EcoRI (New England Biolabs Inc., Ipswich, MA). To construct GST-Splotch Pax3, a linear PCR product was generated using GST-Pax3 FL as template, and primers that would amplify all of the plasmid except nt 839–973 (exon 4). The methylated template was digested with DpnI (New England Biolabs Inc., Ipswich, MA). To generate GST fusion proteins encoding murine Mdm2 and p53, total RNA from whole mouse embryos was reverse transcribed as described [56], and the resulting cDNA was amplified using primer sequences, above. The p53 PCR product was digested with BamHI and EcoRI and inserted into the BamHI and EcoRI sites of pGEX-3X. The Mdm2 PCR product was digested with BglII and EcoRI and inserted into the BamHI and EcoRI sites of pGEX-3X. All plasmids were grown using competent Rosetta cells (Novagen, Madison, WI). DNA sequencing by the Dana-Farber/Harvard Cancer Center DNA Resource Core confirmed the accuracy of cDNA sequences within all recombinant plasmids.

### Transfection of ESC

All transient and stable transfections of stage 1 ESC were performed using Lipofectamine 2000 (Invitrogen) at a concentration of 10 µg/ml. Stable transfectants were selected using 400 µg/ml of G418 (Invitrogen). Transient transfection cultures were terminated 48 h after transfection.

pCMV-Pax3 [8], [47] or empty CMV vector [8] were stably transfected into cells in 35 mm dishes using 1.8 µg plasmid DNA. Recombinant shRNA plasmids targeting *Pax3* mRNA, or empty vector were stably transfected into cells in 35 mm dishes using 2 µg plasmid DNA. To induce shRNA expression, ESC were first differentiated to stage 3, then 1 µg/ml of doxycycline (Clontech) was added to media on day 4 of stage 3 ESC cultures. Cultures were harvested 48 h after doxycycline administration.

Association of FLAG-Pax3 and Pax3 domain fusion proteins with endogenous ESC proteins was studied using transient transfection of ESC grown in 10 cm plates with 24 µg plasmid DNA. The effect of increasing expression of FLAG-Pax3 and Pax3 domain fusion proteins on p53 protein was tested using transient transfection of ESC in 35 mm plates with 0, 1, 2, or 4 µg plasmid DNA.

### Real time RT-PCR

Total RNA was extracted using Ultraspec (Biotecx Laboratories). Real-time RT-PCR was performed in quadruplicate as described using rRNA as the normalization control [57]. Primer and probe sequences for *Pax3* and *p53* were as previously reported [57], [58]. Primers and probes for rRNA and *Nestin* were obtained from PerkinElmer.

### Immunoblot Analyses

Whole cell extracts were prepared and analyzed by immunoblot as described [27], [59]. Antibodies and their working dilutions of primary and secondary antibodies are listed in Table S3. Antibodies coupled to horseradish peroxidase (HRP) were detected by chemiluminescence (PerkinElmer) and exposure to x-ray film. Band intensity was quantified using Adobe Photoshop (Version 9.01).

### Immunoprecipitation

Two hundred µg protein from whole cell extracts were pre-cleared at 4°C for 1 hour with 10 mg non-immune IgG and 10 ml of 50% protein A/G beads (Santa Cruz Biotechnology). The pre-cleared extract was incubated with appropriate antibodies (Table S4) while rocking at 4°C overnight. Protein A/G beads were added for one hour prior to precipitation. The precipitated proteins were analyzed by immunoblot as above.

### Immunofluorescence microscopy

ESC were grown on gelatin-coated cover slips. Cells were fixed with 4% paraformaldehyde, permeabilized with 10% Triton X-100-PBS, blocked in 5% BSA-PBS, and washed in 1% BSA-PBS. The cells were then incubated with primary antibodies at 4°C overnight, and secondary antibodies for 1 hour in the dark at room temperature (see Supplementary Table S5 for antibody details). Cells were counterstained for 5 min. with 300 nM DAPI in PBS. Both antibody incubations were followed by three 10 minutes washes in PBS. Cells were imaged with a Nikon 80i fluorescence microscope.

### Measurement of rates of p53 protein synthesis and decay

p53 synthesis in stage 1 and stage 3 ESC was assayed by pulse labeling with ^35^S-methionine. Briefly, cultures were incubated in cysteine- and methionine-free DMEM for 15 minutes. The media were replaced with cysteine- and methionine-free media containing 0.17 µCi/µl ^35^S-methionine (1175 Ci/mmol) and cultures were incubated for times indicated. p53 was immunoprecipitated from whole cell extracts, electrophoresed, and immunoblotted. p53 bands were cut from nitrocellulose filters and were counted in a scintillation counter (Beckman Coulter). The relative amount of newly synthesized p53 was expressed as ^35^S-cpm/p53 immunoreactivity (determined by scanning and quantitation of x-ray film). Curves were compared using nonlinear regression.

The rates of p53 decay in stage 1 and stage 3 ESC were assayed by pulse-chase labeling. Briefly, cells were labeled with ^35^S-methionine-containing media as above for 1 hour. Media were removed, cultures were rinsed two times with PBS, and were then incubated in complete DMEM. The amount of ^35^S-p53 at each time point was quantitated as above. Nonlinear regression was used to calculate the half-life of p53.

### *In vitro* GST Fusion Protein Association Assay

Expression of GST fusion proteins by Rosetta *E. coli* was induced as described [60]. GST fusion proteins were isolated as described [8]. Five hundred mg protein from stage 1 whole cell lysates were incubated with glutathione-sepharose beads (GE healthcare, Piscataway, NJ) coupled to GST fusion proteins, according to the manufacturer's instructions. The ESC proteins that co-precipitated with GST fusion proteins were identified by immunoblot using antibodies listed in Table S3.

### *In vivo* FLAG Fusion Protein Association Assay

Whole cell lysates were prepared from ESC transiently transfected with FLAG fusion proteins. 500 µg protein were immunoprecipitated with anti-FLAG M2 agarose (Table S4) according to the manufacturer's instructions. The ESC proteins that co-precipitated with FLAG fusion proteins were identified by immunoblot using antibodies listed in Table S3.

### *In vitro* Ubiquitination Assay

*In vitro* ubiquitination reactions were performed as described [60]. The reaction mixture (20 µl) contained 10 ng GST-p53 (murine), 24 ng E1 (Boston Biochem), 20 ng GST-UbcH5C (Boston Biochem), 150 ng GST-Mdm2 (murine), 10 µg His-ubiquitin (Boston Biochem), plus GST fusion proteins containing full length Pax3 or Pax3 domains. After incubation at 37°C for 60 min, the reaction products were terminated with stop buffer (Boston Biochem). Ubiquitinated and unubiquitinated p53 were detected by immunoblot using goat anti-p53 antibodies.

### *In vivo* Ubiquitination Assay

Whole cell lysates were prepared from stage 1 or stage 3 ESC, or from stage 1 ESC transiently transfected for 48 h with 4 µg FLAG-tagged plasmid DNA in 35 mm plates. 500 µg protein were pre-cleared and immunoprecipitated with anti-p53 antibodies (Ab1 and Ab3) and protein A/G beads at 4°C overnight. The precipitated proteins were analyzed by immunoblot with anti-ubiquitin antibodies.

### Statistical Analyses

All statistical analyses were performed using GraphPad Prism software v. 4.0. Data shown are mean ± S.E.M.

## Supporting Information

Figure S1 **Locations of Waardenburg syndrome types 1 and 3 mutations within** ***PAX3*** **.** The major structural domains and locations of intron-exon borders are shown. Mutations causing premature termination are shown above the protein structure, and those that do not cause premature termination (frame-shift or deletion) are shown below the protein structure. Mutations associated with WS3 are shown in italics. Mutations caused by nucleotide insertions or deletions are indicated by nt location and number of inserted or deleted nt; mutations caused by point mutations are indicated by amino acid substitutions. Further description of *PAX3* mutations associated with WS1 and 3 and references are located in Table S6. ***^§^***Patient with WS3 is homozygous for mutation; *2 unrelated families share identical mutations; **^#^**3 unrelated families share identical mutations; **^a^**base substitution at nt +1 within intron 3 abolishes splice donor sequence, causing translation of intron 3 and termination within the PD; **^b^**base substitution at the splice acceptor site of intron 3 causes deletion of exon 4; **^c^**base substitution in the splice donor site of intron 5 causes termination after exon 5. Pax3 structural domains are labeled as in Figure 2.
(TIF)

Table S1 **Oligonucleotide Sequences for Pax3 shRNA.** Short hairpin RNA (shRNA) sequences targeting *Pax3* mRNA were designed and inserted into pSingle-tTS-shRNA (Clontech) as described in Supplementary Materials and Methods. Xho I sites are highlighted in green; short hairpin sequences are highlighted in yellow; Mlu I sites are highlighted in purple; Hind III sites are highlighted in turquoise.
(DOC)

Table S2 **Primer sequences and PCR conditions for construction of GST fusion proteins.** Key: FL, full length; DBD, DNA-binding domains; ID, inhibitory domain; PD, paired domain; OCT, conserved octapeptide; HD, homeodomain. ^*^Genbank accession number. Nucleotides are numbered with “+1” corresponding to the transcription initiation site.
(DOC)

Table S3 **Immunoblot Antibodies.** Antibodies used for immunoblot, their dilutions, species of origin, and commercial sources.
(DOC)

Table S4 **Immunoprecipitation Antibodies.** Antibodies used for immunoprecipitation, amounts used, species of origin, and commercial sources.
(DOC)

Table S5 **Immunofluorescence Antibodies.** Antibodies used for immunoprecipitation, dilutions, species of origin, and commercial sources.
(DOC)

Table S6 **Waardenburg Syndrome Types 1 and 3 Mutations.** Summary of currently identified *PAX3* mutations associated with Waardenburg Syndromes Types 1 and 3 and effects on PAX3 protein.
(DOC)

## References

[1] Robson EJ, He SJ, Eccles MR (2006). A PANorama of PAX genes in cancer and development.. Nat Rev Cancer.

[2] Stuart ET, Kioussi C, Gruss P (1994). Mammalian Pax Genes.. Ann Rev Genet.

[3] Goulding MD, Chalepakis G, Deutsch U, Erselius JR, Gruss P (1991). Pax-3, a novel murine DNA binding protein expressed during early neurogenesis.. EMBO J.

[4] Auerbach R (1954). Analysis of the developmental effects of a lethal mutation in the house mouse.. J Exp Zool.

[5] Bober E, Franz T, Arnold HH, Gruss P, Tremblay P (1994). Pax-3 is required for the development of limb muscles: a possible role for the migration of dermomyotomal muscle progenitor cells.. Development.

[6] Epstein JA, Li J, Lang D, Chen F, Brown CB (2000). Migration of cardiac neural crest cells in Splotch embryos.. Development.

[7] Read AP, Newton VE (1997). Waardenburg syndrome.. J Med Genet.

[8] Phelan S, Loeken M (1998). Identification of a new binding motif for the paired domain of Pax-3 and unusual characteristics of spacing and of bipartite recognition elements on binding and transcription activation.. J Biol Chem.

[9] Chalepakis G, Wijnholds J, Gruss P (1994). Pax-3 DNA interaction-flexibility in the DNA binding and induction of DNA conformational changes by paired domains.. Nuc Acids Res.

[10] Chalepakis G, Gruss P (1995). Identification of DNA recognition sequences for the Pax3 paired domain.. Gene.

[11] Chalepakis G, Goulding M, Read A, Strachan T, Gruss P (1994). Molecular basis of splotch and Waardenburg Pax-3 mutations.. Proc Natl Acad Sci USA.

[12] Watanabe A, Takeda K, Ploplis B, Tachibana M (1998). Epistatic relationship between Waardenburg syndrome genes MITF and PAX3.. Nat Genet.

[13] Mayanil CS, Pool A, Nakazaki H, Reddy AC, Mania-Farnell B (2006). Regulation of murine TGFbeta2 by Pax3 during early embryonic development.. J Biol Chem.

[14] Galibert MD, Yavuzer U, Dexter TJ, Goding CR (1999). Pax3 and regulation of the melanocyte-specific tyrosinase-related protein-1 promoter.. J Biol Chem.

[15] Mayanil CS, George D, Freilich L, Miljan EJ, Mania-Farnell B (2001). Microarray analysis detects novel Pax3 downstream target genes.. J Biol Chem.

[16] Maroto M, Reshef R, Munsterberg AE, Koester S, Goulding M (1997). Ectopic Pax-3 activates MyoD and Myf-5 expression in embryonic mesoderm and neural tissue.. Cell.

[17] Tajbakhsh S, Rocancourt D, Cossu G, Buckingham M (1997). Redefining the genetic heirarchies controlling skeletal myogenesis: Pax-3 and Myf-5 act upstream of MyoD.. Cell.

[18] Epstein JA, Shapiro DN, Chang J, Lam PYP, Maas RL (1996). Pax3 modulates expression of the c-Met receptor during limb muscle development.. Proc Natl Acad Sci USA.

[19] Kwang SJ, Brugger SM, Lazik A, Merrill AE, Wu LY (2002). Msx2 is an immediate downstream effector of Pax3 in the development of the murine cardiac neural crest.. Development.

[20] Hill AL, Phelan SA, Loeken MR (1998). Reduced expression of Pax-3 is associated with overexpression of cdc46 in the mouse embryo.. Development, Genes, and Evolution.

[21] Cai J, Phelan SA, Hill AL, Loeken MR (1998). Identification of Dep-1, a new gene that is regulated by the transcription factor, Pax-3, as a marker for altered embryonic gene expression during diabetic pregnancy.. Diabetes.

[22] Wang Q, Kumar S, Mitsios N, Slevin M, Kumar P (2007). Investigation of downstream target genes of PAX3c, PAX3e and PAX3g isoforms in melanocytes by microarray analysis.. Int J Cancer.

[23] Fenby BT, Fotaki V, Mason JO (2008). Pax3 regulates Wnt1 expression via a conserved binding site in the 5′ proximal promoter.. Biochim Biophys Acta.

[24] Phelan SA, Ito M, Loeken MR (1997). Neural tube defects in embryos of diabetic mice: Role of the Pax-3 gene and apoptosis.. Diabetes.

[25] Morgan SC, Lee H-Y, Relaix F, Sandell L, Lavorse J (2008). Cardiac outflow tract septation failure in Pax3-deficient embryos is due to p53-dependent regulation of migrating cardiac neural crest.. Mech Dev.

[26] Relaix F, Polimeni M, Rocancourt D, Ponzetto C, Schafer BW (2003). The transcriptional activator PAX3-FKHR rescues the defects of Pax3 mutant mice but induces a myogenic gain-of-function phenotype with ligand-independent activation of Met signaling in vivo.. Genes Dev.

[27] Pani L, Horal M, Loeken MR (2002). Rescue of neural tube defects in Pax-3-deficient embryos by p53 loss of function: implications for Pax-3- dependent development and tumorigenesis.. Genes Dev.

[28] Perry P, Sauer S, Billon N, Richardson WD, Spivakov M (2004). A dynamic switch in the replication timing of key regulator genes in embryonic stem cells upon neural induction.. Cell Cycle.

[29] Lee SH, Lumelsky N, Studer L, Auerbach JM, McKay RD (2000). Efficient generation of midbrain and hindbrain neurons from mouse embryonic stem cells.. Nat Biotechnol.

[30] Lendahl U, Zimmerman LB, McKay RD (1990). CNS stem cells express a new class of intermediate filament protein.. Cell.

[31] Underwood TJ, Amin J, Lillycrop KA, Blaydes JP (2007). Dissection of the functional interaction between p53 and the embryonic proto-oncoprotein PAX3.. FEBS Lett.

[32] Sherr CJ, Weber JD (2000). The ARF/p53 pathway.. Curr Opin Genet Dev.

[33] Chalepakis G, Jones FS, Edelman GM, Gruss P (1994). Pax-3 contains domains for transcription activation and transcription inhibition.. Proc Natl Acad Sci, USA.

[34] Goulding M, Sterrer S, Fleming J, Balling R, Nadeau J (1993). Analysis of the Pax-3 gene in the mouse mutant splotch.. Genomics.

[35] Epstein DJ, Vogan KJ, Trasler DG, Gros P (1993). A mutation within intron 3 of the Pax-3 gene produces aberrantly spliced mRNA transcripts in the splotch (Sp) mouse mutant.. Proc Natl Acad Sci USA.

[36] Wiggan O, Taniguchi-Sidle A, Hamel PA (1998). Interaction of the pRB-family proteins with factors containing paired-like homeodomains.. Oncogene.

[37] Bendall AJ, Ding J, Hu G, Shen MM, Abate-Shen C (1999). Msx1 antagonizes the myogenic activity of Pax3 in migrating limb muscle precursors.. Development.

[38] Stamataki D, Kastrinaki M, Mankoo BS, Pachnis V, Karagogeos D (2001). Homeodomain proteins Mox1 and Mox2 associate with Pax1 and Pax3 transcription factors.. FEBS Lett.

[39] Wheat W, Fitzsimmons D, Lennox H, Krautkramer SR, Gentile LN (1999). The highly conserved beta-hairpin of the paired DNA-binding domain is required for assembly of Pax-Ets ternary complexes.. Mol Cell Biol.

[40] Scholl FA, Kamarashev J, Murmann OV, Geertsen R, Dummer R (2001). PAX3 is expressed in human melanomas and contributes to tumor cell survival.. Cancer Res.

[41] Vachtenheim J, Novotna H (1999). Expression of genes for microphthalmia isoforms, Pax3 and MSG1, in human melanomas.. Cell Mol Biol (Noisy-le-grand).

[42] Schulte TW, Toretsky JA, Ress E, Helman L, Neckers LM (1997). Expression of PAX3 in Ewing's sarcoma family of tumors.. Biochem Mol Med.

[43] Gershon TR, Oppenheimer O, Chin SS, Gerald WL (2005). Temporally regulated neural crest transcription factors distinguish neuroectodermal tumors of varying malignancy and differentiation.. Neoplasia.

[44] Harris RG, White E, Phillips ES, Lillycrop KA (2002). The expression of the developmentally regulated proto-oncogene Pax-3 is modulated by N-Myc.. J Biol Chem.

[45] Bardeesy N, Bastian BC, Hezel A, Pinkel D, DePinho RA (2001). Dual inactivation of RB and p53 pathways in RAS-induced melanomas.. Mol Cell Biol.

[46] Vogan K, Bernstein M, Leclerc JM, Brisson L, Brossard J (1993). Absence of p53 gene mutations in primary neuroblastomas.. Cancer Res.

[47] Maulbecker CC, Gruss P (1993). The oncogenic potential of Pax genes.. EMBO J.

[48] Stuart ET, Haffner R, Oren M, Gruss P (1995). Loss of p53 function through PAX-mediated transcriptional repression.. EMBO Journal.

[49] Lynch MP, Capparelli C, Stein JL, Stein GS, Lian JB (1998). Apoptosis during bone-like tissue development in vitro.. J Cell Biochem.

[50] Olaopa M, Zhou HM, Snider P, Wang J, Schwartz RJ (2011). Pax3 is essential for normal cardiac neural crest morphogenesis but is not required during migration nor outflow tract septation.. Dev Biol.

[51] Lin T, Chao C, Saito S, Mazur SJ, Murphy ME (2005). p53 induces differentiation of mouse embryonic stem cells by suppressing Nanog expression.. Nat Cell Biol.

[52] Hong H, Takahashi K, Ichisaka T, Aoi T, Kanagawa O (2009). Suppression of induced pluripotent stem cell generation by the p53-p21 pathway.. Nature.

[53] Kawamura T, Suzuki J, Wang YV, Menendez S, Morera LB (2009). Linking the p53 tumour suppressor pathway to somatic cell reprogramming.. Nature.

[54] Marion RM, Strati K, Li H, Murga M, Blanco R (2009). A p53-mediated DNA damage response limits reprogramming to ensure iPS cell genomic integrity.. Nature.

[55] Utikal J, Polo JM, Stadtfeld M, Maherali N, Kulalert W (2009). Immortalization eliminates a roadblock during cellular reprogramming into iPS cells.. Nature.

[56] Phelan SA, Ito M, Loeken MR (1997). Neural tube defects in embryos of diabetic mice: role of the Pax-3 gene and apoptosis.. Diabetes.

[57] Chang TI, Horal M, Jain S, Wang F, Patel R (2003). Oxidant regulation of gene expression and neural tube development: Insights gained from diabetic pregnancy on molecular causes of neural tube defects.. Diabetologia.

[58] Toda I, Wickham LA, Sullivan DA (1998). Gender and androgen treatment influence the expression of proto-oncogenes and apoptotic factors in lacrimal and salivary tissues of MRL/lpr mice.. Clin Immunol Immunopathol.

[59] Musi N, Fujii N, Hirshman MF, Ekberg I, Froberg S (2001). AMP-activated protein kinase (AMPK) is activated in muscle of subjects with type 2 diabetes during exercise.. Diabetes.

[60] Li M, Luo J, Brooks CL, Gu W (2002). Acetylation of p53 inhibits its ubiquitination by Mdm2.. J Biol Chem.

