# Supplementary figures and images for: Pax3 Stimulates p53 Ubiquitination and Degradation Independent of Transcription
Source: PLoS One. 2011 Dec 28;6(12):e29379. doi: 10.1371/journal.pone.0029379 (PMC3247257; doi:10.1371/journal.pone.0029379)

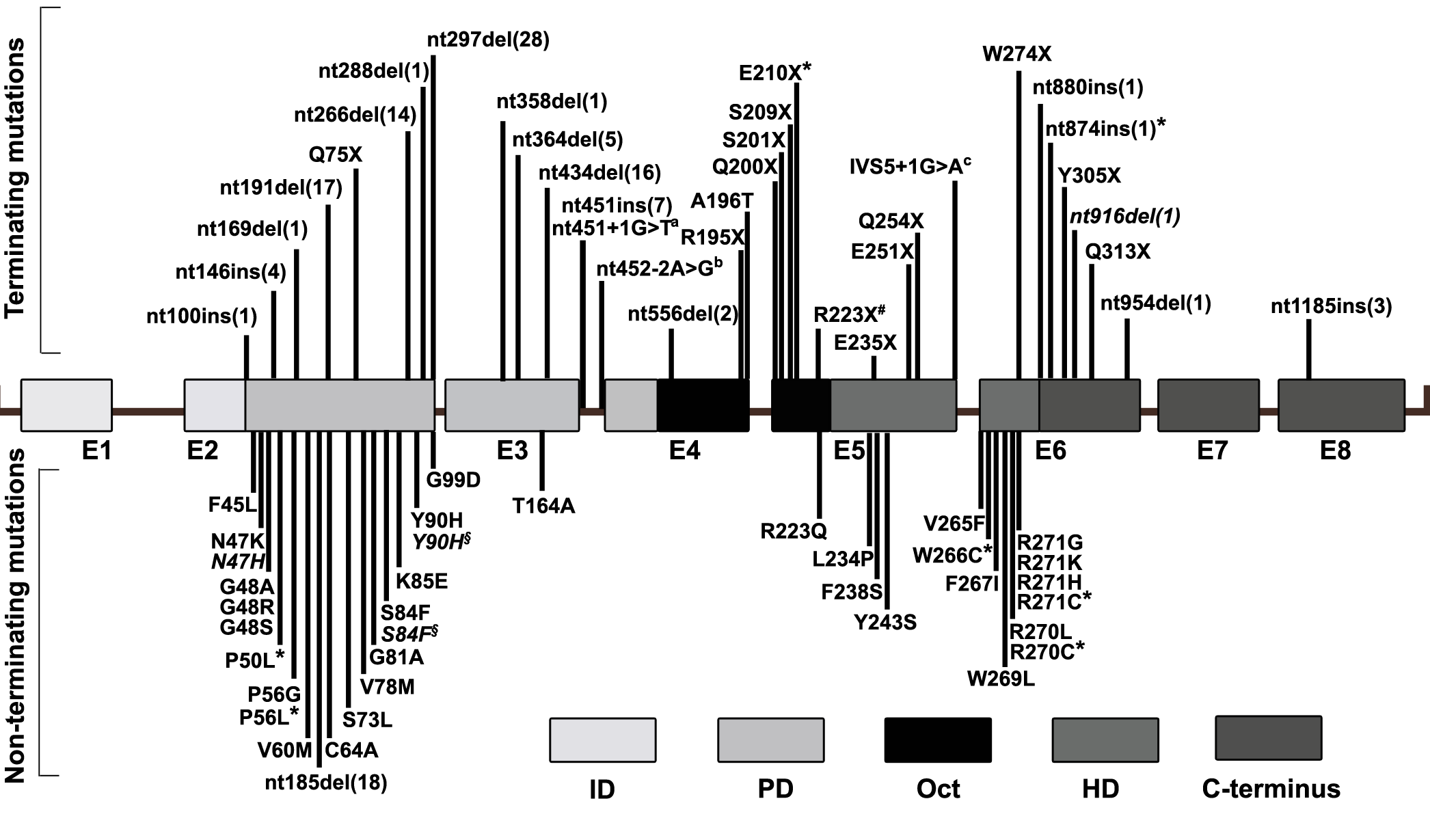

Supplement: Figure S1 — Locations of Waardenburg syndrome types 1 and 3 mutations within PAX3 . The major structural domains and locations of intron-exon borders are shown. Mutations causing premature termination are shown above the protein structure, and those that do not cause premature termination (frame-shift or deletion) are shown below the protein structure. Mutations associated with WS3 are shown in italics. Mutations caused by nucleotide insertions or deletions are indicated by nt location and number of inserted or deleted nt; mutations caused by point mutations are indicated by amino acid substitutions. Further description of PAX3 mutations associated with WS1 and 3 and references are located in Table S6. §Patient with WS3 is homozygous for mutation; *2 unrelated families share identical mutations; #3 unrelated families share identical mutations; abase substitution at nt +1 within intron 3 abolishes splice donor sequence, causing translation of intron 3 and termination within the PD; bbase substitution at the splice acceptor site of intron 3 causes deletion of exon 4; cbase substitution in the splice donor site of intron 5 causes termination after exon 5. Pax3 structural domains are labeled as in Figure 2. (TIF) [file pone.0029379.s001.tif]
