# Supplementary material for: Pax3 Stimulates p53 Ubiquitination and Degradation Independent of Transcription
Source: PLoS One. 2011 Dec 28;6(12):e29379. doi: 10.1371/journal.pone.0029379 (PMC3247257; doi:10.1371/journal.pone.0029379)
Supplement: Table S1 — Oligonucleotide Sequences for Pax3 shRNA. Short hairpin RNA (shRNA) sequences targeting Pax3 mRNA were designed and inserted into pSingle-tTS-shRNA (Clontech) as described in Supplementary Materials and Methods. Xho I sites are highlighted in green; short hairpin sequences are highlighted in yellow; Mlu I sites are highlighted in purple; Hind III sites are highlighted in turquoise. (DOC) [file pone.0029379.s002.doc]

**Table S1. Oligonucleotide Sequences for Pax3 shRNA**

Antisense 1:

Upper strand:

5’ TCGAGGCCCTCAGTGAGTTCTATCATTCAAGAGATGATAGAACTCACTGAG GGTTTTTTACGCGTA 3’

Lower strand:

5’ AGCTTACGCGTAAAAAACCCTCAGTGAGTTCTATCATCTCTTGAATGATAG AACTCACTGAGGGCC 3’

Antisense 2:

Upper strand:

5’ TCGAGGAACCCACTACCCAGACATTTTCAAGAGAAATGTCTGGGTAGTGG GTTTTTTTTACGCGTA 3’

Lower strand:

5’ AGCTTACGCGTAAAAAAAACCCACTACCCAGACATTTCTCTTGAAAATGT CTGGGTAGTGGGTTCC 3’

Antisense 3:

Upper strand:

5’ TCGAGGCAGGTAATGGGACTTCTGATTCAAGAGATCAGAAGTCCCATTAC CTGTTTTTTACGCGTA 3’

Lower strand:

5’ AGCTTACGCGTAAAAAACAGGTAATGGGACTTCTGATCTCTTGAATCAGA AGTCCCATTACCTGCC 3’

Scrambled:

Upper strand:

5’ TCGAGGCCGTACTTGGATGTCAGGAAGGTCATTCAAGAGATGACCTTCCTGACATCCAAGTACGG

TTTTTTACGCGTA 3’

Lower strand:

5’ AGCTTACGCGTAAAAAACCGTACTTGGATGTCAGGAAGGTCATCTCTTGAATGACCTTCCTGACAT

CCAAGTACGGCC 3’
