# Supplementary material for: Pax3 Stimulates p53 Ubiquitination and Degradation Independent of Transcription
Source: PLoS One. 2011 Dec 28;6(12):e29379. doi: 10.1371/journal.pone.0029379 (PMC3247257; doi:10.1371/journal.pone.0029379)
Supplement: Table S2 — Primer sequences and PCR conditions for construction of GST fusion proteins. Key: FL, full length; DBD, DNA-binding domains; ID, inhibitory domain; PD, paired domain; OCT, conserved octapeptide; HD, homeodomain. *Genbank accession number. Nucleotides are numbered with “+1” corresponding to the transcription initiation site. (DOC) [file pone.0029379.s003.doc]

**Table S2. Primer sequences and PCR conditions for construction of GST fusion proteins**

| **Plasmid** | **Primers** | **PCR Condition** | | | |
| --- | --- | --- | --- | --- | --- |
|  |  | **Denaturation temp** | **Annealing temp, time** | **Extension temp, time** | **Cycle #** |
| GST-Pax3 FL  nt 388-1842  NM_001159520^*^ | sense, 5’-TGC GGA TCC GGA TGA CCA CGC TG-3’  antisense, 5’- ACG GAA TTC TCA GCT GTC TTT GCC A -3’ | 94°C | 50°C, 1 min | 72°C, 1.5 min | 30 |
| GST-Splotch Pax3  nt 388-838, 974-1837 | sense, 5'- GAA CAC TGT GCC CTC AGC CTC TGC ACC TCA GTC A -3'  antisense 5'- TGA CTG AGG TGC AGA GGC TGA GGG CAC AGT GTT C -3' | 95°C | 64°C, 1 min | 72°C, 7 min | 30 |
| GST-N-terminus  nt 388-1215 | sense, 5’-TGC GGA TCC GGA TGA CCA CGC TG-3’  antisense, 5’- ACG GAA TTC ATC AGT TGA TTG GCT C -3’ | 94°C | 50°C, 45 s | 72°C, 45 s | 30 |
| GST-C-terminus  nt 1216-1842 | sense, 5’-TGC GGA TCC AGC TGG CCC AGA-3’  antisense, 5’- ACG GAA TTC TCA GCT GTC TTT GCC A -3’ | 94°C | 50°C, 45 s | 72°C, 45 s | 30 |
| GST-DBD  nt 488-1215 | sense, 5’-TGC GGA TCC TGT CCA CCC CTC TT-3’  antisense, 5’- ACG GAA TTC ATC AGT TGA TTG GCT C -3’ | 94°C | 50°C, 45 s | 72°C, 45 s | 30 |
| GST-ID  nt 388-487 | sense, 5’-TGC GGATCC GG ATG ACC ACG CTG GCCGG-3’  antisense, 5'- ACG GAA TTC CCA AGA GGG GTG GAC ACT TCC AG -3' | 94°C | 50°C, 45 s | 72°C for 45 s | 30 |
| GST-PD  nt 488-865 | sense, 5’-TGC GGA TCC TGT CCA CCC CTC TT-3’  antisense, 5'- ACG GAA TTC CTC CTC AGG ATG CGG -3’ | 94°C | 50°C, 45 s | 72°C, 45 s | 30 |
| GST-OCT  nt 866-1045 | sense, 5'- TGC GGA TCC CTA TCA GCC GCA TCC -3'  antisense, 5'- ACG GAA TTC CTC CTG CGC TGC TTC -3' | 94°C | 50°C, 45 s | 72°C, 45 s | 30 |
| GST-HD  nt 1046-1215 | sense, 5'- TGC GGA TCC CTG ATT TAC CGC TGA AG -3'  antisense, 5’- ACG GAA TTC ATC AGT TGA TTG GCT C -3’ | 94°C | 50°C, 45 s | 72°C, 45 s | 30 |
| GST-Mdm2  nt 253-1722  NM_010786^*^ | sense, 5'- TGC AGA TCT CAA TGT GCA ATA CCA ACA TG -3'  antisense, 5'- ACG GAA TTC CAG GTC AGC TAG TTG AAG -3' | 94°C | 50°C, 1 min | 72°C, 1 min | 35 |
| GST-p53  nt 158-1330  NM_011640^*^ | sense, 5'- TGC GGA TCC GGA TGA CTG CCA TGG AG -3'  antisense, 5'- ACG GAA TTC AGT CAG TCT GAG TCA GGC C -3' | 94°C | 50°C, 45 s | 72°C, 45 s | 35 |
