# Supplementary material for: Pax3 Stimulates p53 Ubiquitination and Degradation Independent of Transcription
Source: PLoS One. 2011 Dec 28;6(12):e29379. doi: 10.1371/journal.pone.0029379 (PMC3247257; doi:10.1371/journal.pone.0029379)
Supplement: Table S3 — Immunoblot Antibodies. Antibodies used for immunoblot, their dilutions, species of origin, and commercial sources. (DOC) [file pone.0029379.s004.doc]

**Table S3. Immunoblot Antibodies**

| **Antibody** | **Dilution** | **Species** | **Source** |
| --- | --- | --- | --- |
| anti-Pax3 | 1:1000 | Rabbit | Invitrogen |
| anti-Pax3 | 1:100 | Mouse | Hybridoma Bank |
| anti-p53 (AB1) | 1:500 | Mouse | Calbiochem |
| anti-p53 (AB3) | 1:500 | Mouse | Calbiochem |
| anti-p53 (HRP-coupled) | 1:1000 | Goat | R&D Systems |
| anti-Mdm2 | 1:2000 | Rabbit | R&D Systems |
| anti-Nestin | 1:500 | Goat | Millipore |
| anti-Actin | 1:5000 | Goat | Santa Cruz Biotechnology |
| anti-FLAG | 1:5000 | Rabbit | Sigma |
| anti-mouse IgG (HRP-coupled) | 1:5000 | Donkey | Jackson Immunoresearch Laboratories |
| anti-rabbit IgG (HRP-coupled) | 1:5000 | Donkey | GE Healthcare |
| anti-goat IgG (HRP-coupled) | 1:5000 (1:10000 for actin) | Donkey | Santa Cruz Biotechnology |
| anti-Ubiquitin | 1:800 | Rabbit | Cell Signaling Technology |
