# Supplementary material for: Pax3 Stimulates p53 Ubiquitination and Degradation Independent of Transcription
Source: PLoS One. 2011 Dec 28;6(12):e29379. doi: 10.1371/journal.pone.0029379 (PMC3247257; doi:10.1371/journal.pone.0029379)
Supplement: Table S4 — Immunoprecipitation Antibodies. Antibodies used for immunoprecipitation, amounts used, species of origin, and commercial sources. (DOC) [file pone.0029379.s005.doc]

**Table S4. Immunoprecipitation Antibodies**

| **Antibody** | **Amount** | **Species** | **Source** |
| --- | --- | --- | --- |
| anti-Pax3 | 10 μg | Rabbit | Invitrogen |
| anti-p53 (AB 1) | 5 μg | Mouse | Calbiochem |
| anti-p53 (AB3) | 5 μg | Mouse | Calbiochem |
| anti-Mdm2 | 10 μg | Rabbit | R&D Systems |
| anti-FLAG M2 Agarose | 12 μg | Mouse | Sigma |
