# Supplementary material for: Pax3 Stimulates p53 Ubiquitination and Degradation Independent of Transcription
Source: PLoS One. 2011 Dec 28;6(12):e29379. doi: 10.1371/journal.pone.0029379 (PMC3247257; doi:10.1371/journal.pone.0029379)
Supplement: Table S5 — Immunofluorescence Antibodies. Antibodies used for immunoprecipitation, dilutions, species of origin, and commercial sources. (DOC) [file pone.0029379.s006.doc]

**Table S5. Immunofluorescence Antibodies**

| **Antibody** | **Dilution** | **Species** | **Source** |
| --- | --- | --- | --- |
| anti-Pax3 | 1:100 | Rabbit | Invitrogen |
| anti-p53 (AB1) | 1:100 | Mouse | Calbiochem |
| anti-p53 (AB3) | 1:100 | Mouse | Calbiochem |
| anti-mouse IgG (Alexa-Fluor 488-coupled) | 1:400 | Goat | Invitrogen |
| anti-rabbit IgG (Alexa Fluor 594-coupled) | 1:400 | Goat | Invitrogen |
