# Supplementary material for: Pax3 Stimulates p53 Ubiquitination and Degradation Independent of Transcription
Source: PLoS One. 2011 Dec 28;6(12):e29379. doi: 10.1371/journal.pone.0029379 (PMC3247257; doi:10.1371/journal.pone.0029379)
Supplement: Table S6 — Waardenburg Syndrome Types 1 and 3 Mutations. Summary of currently identified PAX3 mutations associated with Waardenburg Syndromes Types 1 and 3 and effects on PAX3 protein. (DOC) [file pone.0029379.s007.doc]

**Table S6. Waardenburg Syndrome Types 1 and 3 Mutations**

| **Clinical category** | **Family** | **Mutation** | **Location** | **Expected effect on protein** | **Reference** |
| --- | --- | --- | --- | --- | --- |
| **WS1** | WS.018 | del(2)(q34q36.2) | All | Not expressed | [1] |
| **WS1** | WS.055 | F45L | Exon 2 (PD) | Missense mutation at aa45 | [2] |
| **WS1** | WS.005 | nt 185 del(18) | Exon 2 (PD) | Internal deletion within PD, aa62 to 67 | [3,4] |
| **WS1** | WS.100 | nt 191 del(17) | Exon 2 (PD) | Internal deletion within PD, start from aa64, resulting in frameshift mutation: premature termination | [1] |
| **WS1** | WS.024 | V78M | Exon 2 (PD) | Missense mutation at aa78 | [1] |
| **WS1** | WS.015 | G81A | Exon 2 (PD) | Missense mutation at aa81 | [4,5] |
| **WS1** | WS.006 | nt 288 del1 | Exon 3 (PD) | Internal deletion within PD, start from aa96, resulting in frameshift mutation: premature termination | [5] |
| **WS1** | WS.009 | G99D | Exon 2 (PD) | Missense mutation at aa99 | [2] |
| **WS1** | WS.084 | nt 451+1G>T | Intron 3 splice donor | GT –TT at the donor splice site-abolish splicing at this site, resulting a truncated protein | [2] |
| **WS1** | WS.003 | nt 452-2 A>G | Intron 3 splice acceptor | Truncated  AG—GG  at the acceptor splice site, resulting skipping  of exon 4, resulting in a truncated protein | [2] |
| **WS1** | WS.093 | nt 358 del1 | Exon 3 (PD) | Internal deletion within PD, start from aa120, resulting in frameshift mutation: premature termination | [1] |
| **WS1** | WS.138 | A196T | Exon 4 splice donor | Point mutation within PD at aa196, disrupting splicing in exon 4, resulting in a truncated protein | [1] |
| **WS1** | BU26 | P50L | Exon 2 (PD) | Missense mutation at aa50 | [6] |
| **WS1** | BU35 | R56L | Exon 2 (PD) | Missense mutation at aa56 | [6] |
| **WS1** | MSU3 | nt 100 ins1 | Exon 2 (PD) | Internal insertion within PD, start from aa34, resulting in frameshift mutation: premature termination | [7] |
| **WS1** | WGM2 | nt 266 del14 | Exon 2 (PD) | Internal deletion within PD, start from aa87, resulting in frameshift mutation: premature termination | [8] |
| **WS1** | BU53 | nt 297 del28 | Exon3 splice site | Internal deletion within PD, start from aa99, resulting in frameshift mutation: premature termination | [9] |
| **WS1** | BU48 | V60M | Exon 2 (PD) | Missense mutation at aa60 | [10] |
| **WS1** | BU5 | K85E | Exon 2 (PD) | Missense mutation at aa85 | [10] |
| **WS1** | VCU13 | G48R | Exon 2 (PD) | Missense mutation at aa48 | [11] |
| **WS1** | VCU2 | Q75X | Exon 2 (PD) | Nonsense mutation within PD at aa75, resulting in premature termination | [11] |
| **WS1** | UCT44 | nt 451 ins7 | Exon 3 (PD) | Internal insertion within PD, start from aa151, resulting in frameshift mutation: premature termination | [12] |
| **WS1** | WS. 90 | nt 364 del5 | Exon 3 (PD) | Internal deletion within PD, start from aa122, resulting in frameshift mutation: premature termination | [12] |
| **WS1** | VCU52 | nt 169 del1 | Exon 2 (PD) | Internal deletion within PD, start from aa57, resulting in frameshift mutation: premature termination | [11] |
| **WS1** | MSU17 | N47K | Exon 2 (PD) | Missense mutation at aa47 | [13] |
| **WS1** |  | G48A | Exon 2 (PD) | Missense mutation at aa48 | [14] |
| **WS1** |  | G48S | Exon 2 (PD) | Missense mutation at aa48 | [14] |
| **WS1** |  | R56G | Exon 2 (PD) | Missense mutation at aa56 | [14] |
| **WS1** |  | S84F | Exon 2 (PD) | Missense mutation at aa84 | [15] |
| **WS1** |  | nt 364 del5 | Exon 3 (PD) | Internal deletion within PD, start from aa122, resulting in frameshift mutation: premature termination | [16] |
| **WS1** |  | C64A | Exon 1 (PD) | Missense mutation at aa64 | [17] |
| **WS1** |  | T164A | Exon 2 (PD) | Missense mutation at aa164 | [17] |
| **WS1** |  | S73L | Exon 2 (PD) | Missense mutation at aa73 | [18] |
| **WS1** |  | IVS5+1 G>A | Exon 5 splice site | A splice site mutation causes a splicing defect of exon 5, resulting in a truncated protein | [19] |
| **WS1** |  | nt 146 ins4 | Exon 2 (PD) | Internal insertion within PD, start from aa49, resulting in frameshift mutation: premature termination | [19] |
| **WS1** |  | Y90H Heterozygosity | Exon 2 (PD) | Missense mutation at aa90 | [19] |
| **WS1** | WS.011 | nt 556 del2 | Exon 4 (Oct) | Internal deletion within Oct, start from aa186, resulting in frameshift mutation: premature termination | [5] |
| **WS1** | BU7 | Q200X | Exon 5 (Oct) | Nonsense mutation within Oct at aa200, resulting in premature termination | [10] |
| **WS1** | BU4 | S201X | Exon 5 (Oct) | Nonsense mutation within Oct at aa201, resulting in premature termination | [10] |
| **WS1** | VCU3 | E210X | Exon 5 (Oct) | Nonsense mutation within Oct at aa210, resulting in premature termination | [11] |
| **WS1** | VCU56 | E210X | Exon 5 (Oct) | Nonsense mutation within Oct at aa210, resulting in premature termination | [11] |
| **WS1** |  | nt 556 del2 | Exon 4 (Oct) | Internal deletion within Oct, start from aa186, resulting in frameshift mutation: premature termination | [16] |
| **WS1** |  | R195X | Exon 4 (Oct) | Nonsense mutation within Oct at aa195, resulting in premature termination | [16] |
| **WS1** | WS.030 | E251X | Exon 5 (HD) | Nonsense mutation within HD at aa251, resulting in premature termination | [1] |
| **WS1** | WS.001 | Q254X | Exon 5 (HD) | Nonsense mutation within HD at aa254, resulting in premature termination | [1] |
| **WS1** | WS.028 | W266C | Exon 6 (HD) | Missense mutation at aa266 | [1] |
| **WS1** | WS.123 | W274X | Exon 6 (HD) | Nonsense mutation within HD at aa274, resulting in premature termination | [1] |
| **WS1** | WS.016 | R270C | Exon 6 (HD) | Missense mutation at aa270 | [1] |
| **WS1** | WS.008 | R271H | Exon 6 (HD) | Missense mutation at aa271 | [1] |
| **WS1** | WS.010 | R271C | Exon 6 (HD) | Missense mutation at aa271 | [1] |
| **WS1** | BU9 | R223X | Exon 5 (HD) | Nonsense mutation within HD at aa223, resulting in premature termination | [9] |
| **WS1** | BU8 | E235X | Exon 5 (HD) | Nonsense mutation within HD at aa235, resulting in premature termination | [10] |
| **WS1** | BU52 | F238S | Exon 5 (HD) | Missense mutation at aa238 | [10] |
| **WS1** | UCT1 | R223Q | Exon 5 (HD) | Missense mutation at aa223 | [12] |
| **WS1** | VCU29 | R223X | Exon 5 (HD) | Nonsense mutation within HD at aa223, resulting in premature termination | [11] |
| **WS1** | NIH3 | V265F | Exon 6 (HD) | Missense mutation at aa265 | [20] |
| **WS1** | NIH8 | R271G | Exon 6 (HD) | Missense mutation at aa271 | [20] |
| **WS1** | VCU27 | R271K | Exon 6 (HD) | Missense mutation at aa271 | [11] |
| **WS1** | VCU50 | R270C | Exon 6 (HD) | Missense mutation at aa270 | [11] |
| **WS1** | MSU7 | W274X | Exon 6 (HD) | Nonsense mutation within HD at aa274, resulting in premature termination | [21] |
| **WS1** |  | W266C | Exon 6 (HD) | Missense mutation at aa266 | [16] |
| **WS1** |  | W269L | Exon 6 (HD) | Missense mutation at aa269 | [16] |
| **WS1** |  | F267I | Exon 6 (HD) | Missense mutation at aa267 | [22] |
| **WS1** |  | Y243S | Exon 5 (HD) | Missense mutation at aa243 | [23] |
| **WS1** |  | S209X | Exon 5 (HD) | Nonsense mutation within HD at aa209, resulting in premature termination | [24] |
| **WS1** |  | L234P | Exon 5 (HD) | Missense mutation at aa234 | [25] |
| **WS1** | BU14 | Q313X | Exon 6 (C-terminus) | Nonsense mutation within C-terminus at aa313, resulting in premature termination | [10] |
| **WS1** | MSU9 | nt 880 ins1 | Exon 6 (C-terminus) | Internal insertion within C-terminus, start from aa294, resulting in frameshift mutation: premature termination | [12] |
| **WS1** | MSU9 | nt 874 ins1 | Exon 6 (C-terminus) | Internal insertion within C-terminus, start from aa292, resulting in frameshift mutation: premature termination | [21] |
| **WS1** | WS.086 | nt 874 ins1 | Exon 6 (C-terminus) | Internal insertion within C-terminus, start from aa292, resulting in frameshift mutation: premature termination | [1] |
| **WS1** | BU22 | nt 874 ins1 | Exon 6 (C-terminus) | Internal insertion within C-terminus, start from aa292, resulting in frameshift mutation: premature termination | [10] |
| **WS1** | BU30 | nt 954 del1 | Exon 6 (C-terminus) | Internal deletion within C-terminus, start from aa318, resulting in frameshift mutation: premature termination | [10] |
| **WS1** | BU25 | nt 1185 ins3 | Exon 8 (C-terminus) | Internal insertion within C-terminus, start from aa395, resulting in frameshift mutation: premature termination | [10] |
| **WS1** |  | Y305X | Exon 6 (C-terminus) | Nonsense mutation within C-terminus at aa305, resulting in premature termination | [16] |
| **WS3** |  | S84F Homozygosity | Exon 2 (PD) | Missense mutation at aa84 | [15] |
| **WS3** |  | nt 434 del16 | Exon 3 (PD) | Internal deletion within PD, start from aa147, resulting in frameshift mutation: premature termination | [16] |
| **WS3** | BU47 | N47H | Exon 2 (PD) | Missense mutation at aa47 | [6] |
| **WS3** |  | Y90H Homozygosity | Exon 2 (PD) | Missense mutation at aa90 | [19] |
| **WS3** | WS.105 | nt 916del1 | Exon 6 (HD) | Internal deletion within HD, start from aa306, resulting in frameshift mutation: premature termination | [1] |
| **WS3** |  | del (2)(q35q36) |  | Not expressed | [26] |

**REFERENCES**

1. Tassabehji M, Newton VE, Liu XZ, Brady A, Donnai D, et al. (1995) The mutational spectrum in Waardenburg syndrome. Hum Mol Genet 4: 2131-2137.

2. Tassabehji M, Newton VE, Leverton K, Turnbull K, Seemanova E, et al. (1994) PAX3 gene structure and mutations: close analogies between Waardenburg syndrome and the Splotch mouse. Hum Mol Genet 3: 1069-1074.

3. Tassabehji M, Read AP, Newton VE, Harris R, Balling R, et al. (1992) Waardenburg's syndrome patients have mutations in the human homologue of the Pax-3 paired box gene. Nature 355: 635-636.

4. Chalepakis G, Goulding M, Read A, Strachan T, Gruss P (1994) Molecular basis of splotch and Waardenburg Pax-3 mutations. Proc Natl Acad Sci U S A 91: 3685-3689.

5. Tassabehji M, Read AP, Newton VE, Patton M, Gruss P, et al. (1993) Mutations in the PAX3 gene causing Waardenburg syndrome type 1 and type 2. Nat Genet 3: 26-30.

6. Hoth CF, Milunsky A, Lipsky N, Sheffer R, Clarren SK, et al. (1993) Mutations in the paired domain of the human PAX3 gene cause Klein-Waardenburg syndrome (WS-III) as well as Waardenburg syndrome type I (WS-I). Am J Hum Genet 52: 455-462.

7. Morell R, Friedman TB, Asher JH, Jr. (1993) A plus-one frameshift mutation in PAX3 alters the entire deduced amino acid sequence of the paired box in a Waardenburg syndrome type 1 (WS1) family. Hum Mol Genet 2: 1487-1488.

8. Morell R, Friedman TB, Moeljopawiro S, Hartono, Soewito, et al. (1992) A frameshift mutation in the HuP2 paired domain of the probable human homolog of murine Pax-3 is responsible for Waardenburg syndrome type 1 in an Indonesian family. Hum Mol Genet 1: 243-247.

9. Baldwin CT, Lipsky NR, Hoth CF, Cohen T, Mamuya W, et al. (1994) Mutations in PAX3 associated with Waardenburg syndrome type I. Hum Mutat 3: 205-211.

10. Baldwin CT, Hoth CF, Macina RA, Milunsky A (1995) Mutations in PAX3 that cause Waardenburg syndrome type I: ten new mutations and review of the literature. Am J Med Genet 58: 115-122.

11. Pandya A, Xia XJ, Landa BL, Arnos KS, Israel J, et al. (1996) Phenotypic variation in Waardenburg syndrome: mutational heterogeneity, modifier genes or polygenic background? Hum Mol Genet 5: 497-502.

12. DeStefano AL, Cupples LA, Arnos KS, Asher JH, Jr., Baldwin CT, et al. (1998) Correlation between Waardenburg syndrome phenotype and genotype in a population of individuals with identified PAX3 mutations. Hum Genet 102: 499-506.

13. Asher JH, Jr., Sommer A, Morell R, Friedman TB (1996) Missense mutation in the paired domain of PAX3 causes craniofacial-deafness-hand syndrome. Hum Mutat 7: 30-35.

14. Fortin AS, Underhill DA, Gros P (1997) Reciprocal effect of Waardenburg syndrome mutations on DNA binding by the Pax-3 paired domain and homeodomain. Hum Mol Genet 6: 1781-1790.

15. Zlotogora J, Lerer I, Bar-David S, Ergaz Z, Abeliovich D (1995) Homozygosity for Waardenburg syndrome. Am J Hum Genet 56: 1173-1178.

16. Read AP, Newton VE (1997) Waardenburg syndrome. J Med Genet 34: 656-665.

17. Ptok M, Morlot S (2006) [Unilateral sensineural deafness associated with mutations in the PAX3-gene in Waardenburg syndrome type I]. HNO 54: 557-560.

18. Sotirova VN, Rezaie TM, Khoshsorour MM, Sarfarazi M (2000) Identification of a novel mutation in the paired domain of PAX3 in an Iranian family with waardenburg syndrome type I. Ophthalmic Genet 21: 25-28.

19. Wollnik B, Tukel T, Uyguner O, Ghanbari A, Kayserili H, et al. (2003) Homozygous and heterozygous inheritance of PAX3 mutations causes different types of Waardenburg syndrome. Am J Med Genet A 122A: 42-45.

20. Lalwani AK, Brister JR, Fex J, Grundfast KM, Ploplis B, et al. (1995) Further elucidation of the genomic structure of PAX3, and identification of two different point mutations within the PAX3 homeobox that cause Waardenburg syndrome type 1 in two families. Am J Hum Genet 56: 75-83.

21. Morell R, Carey ML, Lalwani AK, Friedman TB, Asher JH, Jr. (1997) Three mutations in the paired homeodomain of PAX3 that cause Waardenburg syndrome type 1. Hum Hered 47: 38-41.

22. Nakamura M, Ishikawa O, Tokura Y (2009) A novel missense mutation in the PAX3 gene in a case of Waardenburg syndrome type I. J Eur Acad Dermatol Venereol 23: 708-709.

23. Kozawa M, Kondo H, Tahira T, Hayashi K, Uchio E (2009) Novel mutation in PAX3 gene in Waardenburg syndrome accompanied by unilateral macular degeneration. Eye 23: 1619-1621.

24. Yang SZ, Cao JY, Zhang RN, Liu LX, Liu X, et al. (2007) Nonsense mutations in the PAX3 gene cause Waardenburg syndrome type I in two Chinese patients. Chin Med J (Engl) 120: 46-49.

25. Qin W, Shu A, Qian X, Gao J, Xing Q, et al. (2006) A novel mutation of PAX3 in a Chinese family with Waardenburg syndrome. Mol Vis 12: 1001-1008.

26. Pasteris NG, Trask BJ, Sheldon S, Gorski JL (1993) Discordant phenotype of two overlapping deletions involving the PAX3 gene in chromosome 2q35. Hum Mol Genet 2: 953-959.
